# A Scientific Validation of Antihyperglycemic and Antihyperlipidemic Attributes of *Trichosanthes dioica*

**DOI:** 10.1155/2013/473059

**Published:** 2013-07-25

**Authors:** Prashant Kumar Rai, Sharad Kumar Gupta, Amrita Kumari Srivastava, Rajesh Kumar Gupta, Geeta Watal

**Affiliations:** ^1^Alternative Therapeutics Unit, Drug Development Division, Medicinal Research Laboratory, Department of Chemistry, University of Allahabad, Allahabad 211 002, India; ^2^Department of NMR, All India Institute of Medical Sciences, New Delhi 110 029, India; ^3^School of Vocational Studies and Applied Chemistry, Gautam Buddha University, Greater Noida, Uttar Pradesh 201310, India

## Abstract

The present study was undertaken to scientifically validate the antidiabetic activity of aqueous fruit extract of *Trichosanthes dioica* Roxb. (Family: Cucurbitaceae) which has been traditionally used for managing diabetes mellitus. This plant commonly known as “Sespadula” in English has not been explored scientifically so far for its glycemic potential except by our research group. The study was conducted with variable doses on normal, mild, and severe diabetics models, and several biochemical parameters including blood glucose level (BGL) were assessed. Maximum fall in BGL of 23.8% in normal rats and of 31.3% in mild diabetic rats was observed during their fasting blood glucose (FBG) and glucose tolerance test (GTT) with the dose of 1000 mg kg^−1^. In severely diabetic animals after 4 weeks treatment with FBG, postprandial glucose, total cholesterol, and triglyceride levels were reduced by 28.7, 30.7, 57.2, and 18.5%, whereas high density lipoprotein, total protein, hemoglobin, and body weight were increased by 33.0, 36.7, 15.7 and 16.7%, respectively. Moreover, urine sugar was reduced from +4 to +1. Thus, the study scientifically validates the traditional use of *T. diocia* in diabetes management and could be developed as an effective oral agent for treating diabetes mellitus and complications associated with it.

## 1. Introduction

Diabetes mellitus and its complications are becoming a global burden and have to be dealt with firmly. Hypercholesterolemia associated with this dreaded disease [1, 2] has been ranked as one of the greatest risk factors contributing to the prevalence and severity of coronary heart diseases [3]. World Health Organization (WHO) has recommended the development of oral hypoglycemic agents from medicinal plants [4] as herbal natural remedies to treat diabetes mellitus being cost-effective and safe [5]. Many plants have been explored scientifically and systematically and proved to be beneficial for the treatment of diabetes mellitus by our research group [6–10]. The present study is a further effort in the direction of developing a novel, oral antidiabetic agent coupled with antilipidemic efficacy of high potential with minimal or no side effects. 

*Trichosanthes dioica *(*T. dioica*) Roxb. (Family: Cucurbitaceae) is commonly known as “Sespadula” in English and “Parwal” in Hindi and is widely grown throughout India [11]. Its fruits are used as vegetable from the time immemorial and have also been proved as hypocholesterolemic and hypoglyceridimic in case of normal animals after shade drying and mixing in the food [12]. Recently its seeds and leaves have also been experimentally proven as antidiabetic agents by our research group [13, 14]. Phytochemical investigation of its fruits and seeds reveal the presence of all those classes of compounds which are responsible either for managing diabetes or its complications, namely, flavonoids [15, 16], alkaloids [17–22], glycosides [23, 24], terpenes, and sterols [25–27]. This plant also serves as a rich source of minerals such as Mg, Na, K, Cu, and S [28] whose significant role in controlling and managing diabetes is well known and cannot be ignored as specific concentration of these minerals have been reported to take part in carbohydrate metabolism as well as insulin release [29–32]. The present study was designed to scientifically validate the use of *T. dioica* in folklore medicines for treating diabetes by evaluating its glycemic potential. The effect was observed on blood glucose level (BGL) of normal and streptozotocin- (STZ-) induced mild and severely diabetic rats with graded doses. Biochemical parameters such as fasting blood glucose (FBG) in addition to lipid profile and other parameters were considered. Results of all valuable scientific measures taken into consideration reveal that along with antidiabetic and antilipidemic activities it has high nutritive value also as the plant has been well known since ages for regaining health post sickness [33].

## 2. Materials and Methods

### 2.1. Plant Material

Fresh unripe fruits (6 kg) of *T. dioica* were purchased from the local market of Allahabad, U P (India) and authenticated by Professor Satya Narayan, Taxonomist, Department of Botany, University of Allahabad, Allahabad, U P, India. A voucher specimen (AA512) has been submitted to the University herbarium. The fruits were cut into small pieces and shade dried. The dried pieces were mechanically crushed and extracted with distilled water using soxhlet at temperature (80–100°C) up to 36 h. The extract was filtered and concentrated in rotatory evaporator at 35 ± 5°C under reduced pressure, to obtain semisolid material, which was then lyophilized to get a powder (yield: 14.9% w/w).

### 2.2. Experimental Animals

Experiments were performed in 6–8-week-old, healthy, male albino Wistar rats, of body weight 150–200 g. Animals obtained from National Institute of Communicable Diseases (NICD) New Delhi, India were housed under standard environmental conditions (25 ± 2°C temperature, 50 ± 5% humidity with a 12 h each of dark and light cycle) and maintained with free access to water and a standard laboratory diet (carbohydrates; 30%, proteins; 22%, lipids; 12%, and vitamins; 3%) *ad libitum*. The study was approved by the Institutional Ethical Committee.

### 2.3. Induction of Diabetes

Diabetes was induced by a single intraperetonial injection of freshly prepared Streptozotocin (STZ) (purchased from Sigma Aldrich Chem. Co., St. Louis, USA) at a standard dose of 55 mg kg^−1^ bw [34] in 0.1 M citrate buffer (pH 4.5) to a group of overnight fasted rats. After 3 days of STZ administration, depending on their glucose levels, the animals were divided into two groups, mild and severely diabetic animals as follows:

- Mild diabetic (MD): FBG 150–250 mg dL^−1^
- Severely diabetic (SD): FBG > 250 mg dL^−1^.

### 2.4. Estimation

Blood samples were collected from the tail vein from the afore mentioned overnight fasted rats, and the BGL were estimated by glucose oxidase method [35] using standard kit of Bayer Diagnostics India Limited, New Delhi, India. TC, TG, and HDL levels in serum were measured spectrophotometrically by the method prescribed by manufacturer [36, 37] using standard kit from Bayer Diagnostics India Limited, New Delhi, India. However, very low density lipoprotein (VLDL) and low density lipoprotein (LDL) cholesterol were calculated by Friedwald's formula, [VLDL = TG/5 and LDL = TC − (VLDL + HDL)] [38]. Total protein (TP) [39] and hemoglobin (Hb) [40] were also estimated in blood before and after the treatment of four weeks duration. Urine sugar (US) was detected by reagent based Uristix from Bayer Diagnostics India Limited, New Delhi, India. All the parameters inclusive of bw were measured initially before the treatment and then monitored regularly every week up to 4 weeks treatment.

### 2.5. Experimental Design

Initial screening of the aqueous extract for the hypoglycemic activity was done with a range of variable doses in overnight fasted normal healthy rats by conducting FBG and glucose tolerance test (GTT) studies. The antidiabetic effect was assessed in mild-diabetic models fasted overnight with the same range of doses by studying their effect on FBG and GTT levels. The results were compared with a reference drug, Tolbutamide, for positive control. Severely diabetic animals were used for evaluating the antidiabetic and hypolipidemic potential of the most effective dose identified in case of normal and mild-diabetic animals. Effect of this dose was also studied on TC, TG, HDL, TP, Hb, US, and bw of severely diabetic rats.

### 2.6. Assessment of Hypoglycemic Activity in Normal Healthy Rats

Five groups of six rats each were used in the experiment. Group I served as untreated control received vehicle (distilled water only), and animals of groups II, III, IV, and V received aqueous fruit extract suspended in distilled water at doses 500, 750, 1000, and 1250 mg kg^−1^ bw, respectively. Blood samples were collected from tail vein at 1.5, 3, 4.5, and 6 h after administering the extract. 

### 2.7. Assessment of Antidiabetic Activity by GTT in Mild-Diabetic Rats

The antidiabetic effect of aqueous extract of *T. dioica *fruits in mild-diabetic rats was also assessed for improvement in glucose tolerance. The rats were divided into six groups. Group I served as control, received vehicle (distilled water only), whereas variable doses of 500, 750, 1000, and 1250 mg kg^−1^ bw of fruit extract was given orally to groups II, III IV, and V, respectively. BGLs were checked first after 90 minutes of treatment considered as “0” h value and then 2 g kg^−1^ bw glucose was given orally to all the groups. BGLs were further checked up to three hours at regular intervals of 1 h each, considered as 1, 2, and 3 h values. The results were compared with group VI of rats, which were treated with 250 mg kg^−1^ bw of Tolbutamide (hypoglycemic agent) as a reference drug for positive control.

### 2.8. Assessment of Antidiabetic Activity in Severely Diabetic Rats

Three groups of 6 rats each were used in the experiment. Groups I and II served as normal and severely diabetic control, whereas group III was treated once daily for 4 weeks with the dose of 1000 mg kg^−1^ bw identified as the most effective dose in case of normal and mild-diabetic rats. Various biochemical parameters such as FBG, PPG, TC, TG, HDL, LDL, VLDL, TP, Hb, US, and bw were taken into consideration and were estimated initially and then weekly up to 4 weeks.

### 2.9. LD_50_ Experiment

Toxic effect of the aqueous extract was also studied by LD_50_ experiment. Two groups of rats of both the sexes (6 animals per group, 3 females and 3 males), weighing about 180–200 g were orally administered ten and fifteen times the most effective dose of 1000 mg kg^−1^ bw by a single dose of 10.0 g and 15.0 g. of the aqueous extract of *T. dioica *fruits. Thereafter, rats were observed for gross behavioral, neurologic, autonomic, and toxic effects continuously for 24 h. Food consumption, faeces, and urine were also examined at 2 h and then at 6 h intervals for 24 h. 

### 2.10. Statistical Analysis

Data were statistically evaluated using one-way ANOVA (Analysis of Variance), followed by a post hoc Newman-Keuls Multiple Comparison Test. The values were expressed as mean ± S.D. and considered significant at *P* < 0.05. 

## 3. Results

### 3.1. Effect on FBG of Normal Healthy Rats

Table 1 describes the hypoglycemic effect of a single oral administration of variable doses of 500, 750, 1000, and 1250 mg kg^−1^ bw of aqueous fruit extract in normal healthy rats. Treated rats showed a regular fall of 14.0, 18.2, and 23.8% (*P* < 0.01) from the doses of 500, 750, and 1000 mg kg^−1^ bw, respectively, after 6 h. However, a fall of only 16.2% (*P* < 0.01) was observed with the dose of 1250 mg kg^−1^ after the same interval of time. 

### 3.2. Effect on GTT of Mild-Diabetic Rats

Table 2 illustrates the antidiabetic effect of single dose treatment of different doses of extract as mentioned previously and also of a dose of 250 mg kg^−1^ bw Tolbutamide (standard reference drug). The fall of 16.4, 29.7, 31.3, and 28.6% (*P* < 0.01) in BGL of mild-diabetic animals was observed after 3 h of glucose administration with the doses of 500, 750, 1000, and 1250 mg kg^−1^ bw, respectively. However, the dose of 250 mg kg^−1^ bw of Tolbutamide reduced BGL by 31.5% (*P* < 0.01) at 3 h during GTT in mild-diabetic rats, which is almost at par with the most effective dose of 1000 mg kg^−1^ bw of the aqueous extract. 

### 3.3. Effect on FBG, PPG, and Lipid Profile of Severely Diabetic Rats

Tables 3 and 4 describe the antidiabetic and antilipidemic effect of long-term treatment of 4 weeks with the dose of 1000 mg kg^−1^ bw of the extract on BGL and lipid profile of severely diabetic rats. Rats were treated with the most effective dose of 1000 mg kg^−1^ bw of the aqueous extract once a day at noon for four weeks. At the end of the treatment, the animals were compared with their own initial values and showed a significant reduction of 28.7% (*P* < 0.001) in FBG and 30.7% (*P* < 0.001) in PPG levels. The enhanced levels of TC and TG prior to the treatment, were brought down significantly to 57.2 and 18.5%, (*P* < 0.01), respectively, after the treatment period. There was also significant improvement of 33.0% (*P* < 0.05) observed in HDL level of severely diabetic treated group. The values calculated for LDL and VLDL from the previous data using Friedwald's formula also showed a marked reduction of 9.6 and 57.2% (*P* < 0.05), respectively, in their levels. 

### 3.4. Effect on TP and Hb US and BW of Severely Diabetic Rats

Tables 5 and 6 demonstrate the effect of afore mentioned long-term treatment of the aqueous extract on TP, Hb, US, and bw. The unique observation was a potential improvement of 36.7 and 15.7% (*P* < 0.05) in TP and Hb levels of severely diabetic rats, respectively. In addition, 75% (*P* < 0.05) decrease in urine sugar levels and 16.7% (*P* < 0.05) increase in body weight was observed. Contrarily, enhanced levels of urine sugar accompanied with weight loss were observed in severely diabetic control group.

### 3.5. LD_50_ Experiment

Experiment was carried out on normal healthy rats. The behavior of the treated rats appeared normal. No toxic effect was reported at doses up to 10 and 15 times of the identified most effective dose of the aqueous extract as no mortality was observed in any of these groups. 

## 4. Discussion

Fruits of *T. dioica* have been used as a revitalizing agent since ages in the Indian food system as well as in Ayurvedic system of medicine [33]. Since its glycemic and lipidemic profiles have never been validated scientifically so far therefore the present study was undertaken in normal, mild, and severely diabetic models with variable doses. Moreover, the dose of 1000 mg kg^−1^ bw produced maximum fall in BGL of 23.8% within 6 h during FBG studies, whereas the GTT studies of mild-diabetic animals showed marked improvement of 31.3% in glucose tolerance within 3 h suggesting thereby that the active ingredients of the aqueous extract or their metabolites may take about 180 minutes to exhibit their hypoglycemic effect by reaching the target tissues through circulation. 

The results obtained with the dose of 250 mg kg^−1^ bw of the reference drug, Tolbutamide, taken as standard are comparable with the results of the most effective dose of 1000 mg kg^−1^ bw of the extract indicating thereby the possible similar mechanism of action [41, 42]. Thus, it may be the plausible mechanism by which the aqueous extract is able to decrease blood sugar level by increasing the pancreatic secretion of insulin from beta cells of islets of Langerhans [43].

Generally, it has been observed that hyperlipidemia is a complication associated with hyperglycemia [44] and the most common lipid abnormality observed is hypertriglyceridemia which increases risk factor of strokes [45]. Therefore, ideal treatment of diabetes, in addition to glycemic control, should have a favourable effect on lipid profiles [43]. 

Results reveal that *T. dioica* aqueous fruit extract evoked significant reductions in TC, TG, LDL, and VLDL levels of severely diabetic rats at the end of 28 days of treatment as compared to the initial. It has been found that most of the drugs that decrease TC also decrease HDL cholesterol [34] (Singh et al., 2008) but it is noteworthy to mention here that *T. dioica* had an additional advantage over the existing drugs in the way that it not only decreased the TC but also increased the cardioprotective HDL cholesterol after four weeks treatment indicating thereby that the aqueous extract of *T. dioica* fruits possesses considerable efficacy to manage elevated BGL as an aftermath of diabetes in addition to reverting the disturbed lipid profile associated with diabetes. Thus, we may say that *T. dioica* possesses modulatory effects on blood lipid abnormalities associated with diabetes. However, diabetic rats treated with the *T. dioica *aqueous extract showed an increase in body weight as compared to the diabetic control which may be attributed to its protective effect in controlling muscle wasting, that is, reversal of gluconeogenesis. Reversal of gluconeogenesis could be due to improved insulin secretion and glycemic control. Keeping in mind the herbal therapy with the *T. dioica *aqueous fruit extract as an alternative treatment for diabetes, toxicity was also evaluated by LD_50_ experiment. High value of LD_50_ for *T. dioica* implies its great margin of safety. 

A number of lectins have been reported from *T. dioica* [46, 47]. Lactines are responsible for various erythrocyte surface alterations in diabetic cases which result in impaired cell function therefore the improvement in diabetic condition on extract treatment could also be due to the presence of lectins in *T. dioica* extract along with other active components responsible for its antidiabetic and antilipidemic activity. 

## 5. Conclusions

In conclusion, the relevance and significance of this pioneer study cannot be ignored as it reconfirms the ethnobotanical profile of *T. dioica* fruits since the aqueous extract of the plant's fruits helps in reducing the raised levels of BGL, TC, TG, LDL, VLDL, and US procured during diabetic condition. Moreover, the extract proved beneficial in enhancing the low levels of HDL, TP, and Hb at the end of the treatment. Significant increase in bw was an additional advantage. The extract may therefore be of considerable value to human subjects due to its high LD_50_, showing great margin of safety. It can thus be concluded that this plant extract promises an effective breakthrough in its potential development as a powerful oral therapeutic agent for controlling and managing diabetes mellitus. 

## Figures and Tables

**Table 1:** Effect of graded dose of *Trichosanthes dioica* fruit aqueous extract on FBG of normoglycemic rats (mean ± S.D.).

Experimental Groups	Treatment (mg kg^−1^ bw)	Blood glucose levels (mg dL^−1^)				
		Pretreatment	Post treatment (hours)			
		FBG	1.5	3.0	4.5	6.0
Control (I)	Distilled water	69.5 ± 3.9	69.3 ± 3.2	70.0 ± 4.6	70.1 ± 3.8	69.2 ± 4.2
Extract (II)	500	70.0 ± 3.2	68.8 ± 4.4	66.1 ± 4.6	62.4 ± 5.1*	60.1 ± 3.8
Extract (III)	750	68.2 ± 3.2	65.9 ± 4.4	62.1 ± 4.6	58.7 ± 5.1*	55.6 ± 3.8
Extract (IV)	1000	71.4 ± 3.2	68.1 ± 4.4	63.5 ± 4.6	58.9 ± 5.1**	54.3 ± 3.8*
Extract (V)	1250	72.1 ± 4.6	70.4 ± 4.2	67.1 ± 4.8	65.8 ± 3.7**	60.4 ± 4.5*

*P < 0.05, **P < 0.01 compared with control.

**Table 2:** Effect of graded doses of *T. dioica* fruits aqueous extract on BGL during GTT in mild-diabetic rats each value shown in Mean ± S.D. (*n* = 6).

Experimental Groups	Treatment (mg kg^−1^ bw)	Blood glucose levels (mg dL^−1^)				
		Pretreatment	Post treatment (hours)			
		FBG	0	1	2	3
Control (I)	Distilled water	175.4 ± 2.6	173.8 ± 3.2	391.2 ± 4.1	317.4 ± 3.4	280.5 ± 4.2
Extract (II)	500	174.8 ± 4.6	169.2 ± 4.4	329.3 ± 2.9	270.4 ± 3.5	234.3 ± 2.9
Extract (III)	750	168.4 ± 3.5	165.4 ± 3.5	314.5 ± 3.1	252.6 ± 4.1	197 ± 3.7
Extract (IV)	1000	166.6 ± 2.9	151.3 ± 4.6	258.5 ± 3.3	217.4 ± 4.4*	192.4 ± 3.2**
Extract (V)	1250	170.3 ± 3.9	163.4 ± 3.8	309.6 ± 4.6	250.7 ± 3.9*	200.1 ± 4.2*
Tolbutamide (VI)	250	171.2 ± 4.8	159.3 ± 3.6	259.5 ± 4.1	212.4 ± 3.2	192.1 ± 3.4**

*P < 0.05, **P < 0.01 compared to the control at the corresponding time.

**Table 3:** Effect of most effective dose of *Trichosanthes dioica* fruit aqueous extract on BGL of severely diabetic rats (mean ± S.D.).

Experimental animals	Treatment (aqueous extract)	Pretreatment levels	Posttreatment levels			
			7 days	14 days	21 days	28 days
FBG (mg dL^−1^)						
Normal (control I)	D W	82.2 ± 3.5	85.6 ± 2.8	84.6 ± 4.6	83.9 ± 4.8	85.2 ± 3.9
SD (control II)	D W	287.7 ± 5.7	290.9 ± 3.4	302.4 ± 4.6	299.9 ± 4.9	298.7 ± 4.9
SD (treated III)	1000 mg kg^−1^	298.2 ± 7.5	274.3 ± 4.6*	256.5 ± 4.5**	224.41 ± 4.9	212.3 ± 5.2

PPG (mg dL^−1^)						
Normal (control I)	D W	160.8 ± 5.2	162.8 ± 4.7	163.2 ± 4.9	162.1 ± 5.1	161.8 ± 4.1
SD (control II)	D W	447.7 ± 4.8	456.1 ± 5.1	458.9 ± 3.6	452.4 ± 4.5	462.8 ± 3.8
SD (treated III)	1000 mg kg^−1^	438.2 ± 3.5	386.3 ± 5.2**	354.6 ± 4.1*	322.2 ± 4.3*	303.3 ± 4.5**

**P< 0.01, *P< 0.05 compared to pretreatment levels.

**Table 4:** Effect of most effective dose of *Trichosanthes dioica* fruit aqueous extract on lipid profile of severely diabetic rats (mean ± S.D.).

Experimental animals	Treatment (aqueous extract)	Pretreatment levels	Posttreatment levels			
			7 days	14 days	21 days	28 days
Total cholesterol (mg dL^−1^)						
Normal (control I)	D W	95.8 ± 6.4	97.2 ± 6.8	98.7 ± 5.9	98.8 ± 7.4	97.5 ± 6.8
SD (control II)	D W	118.6 ± 4.4	116.4 ± 5.6	114.9 ± 5.2	113.5 ± 4.6	115.3 ± 5.5
SD (treated III)	1000 mg kg^−1^	116.4 ± 5.8	101.2 ± 4.6**	99.7 ± 7.5*	93.6 ± 5.2*	94.7 ± 5.2***

HDL cholesterol (mg dL^−1^)						
Normal (control I)	D W	28.5 ± 4.2	29.8 ± 6.5	30.5 ± 4.6	29.9 ± 5.9	28.7 ± 6.6
SD (control II)	D W	24.8 ± 2.8	24.5 ± 3.2	22.6 ± 8.6	22.1 ± 6.9	22.5 ± 7.6
SD (treated III)	1000 mg kg^−1^	21.0 ± 3.5	25.2 ± 4.9*	25.3 ± 5.1*	27.9 ± 5.6***	28.0 ± 6.8**

Triglycerides (mg dL^−1^)						
Normal (control I)	D W	90.9 ± 4.8	91.1 ± 5.2	91.9 ± 5.4	92.1 ± 8.6	92.6 ± 8.6
SD (control II)	D W	199.2 ± 5.2	197.8 ± 4.8	180.1 ± 5.7	191.3 ± 5.9	199.6 ± 6.2
SD (treated III)	1000 mg/kg	203.8 ± 3.2	161.4 ± 4.4*	101.8 ± 6.1***	89.3 ± 5.7*	87.1 ± 5.7*

***P< 0.001, **P< 0.01, *P< 0.05 as compared to pretreatment levels.

LDL and VLDL cholesterol (mg dL^−1^) were calculated with Friedwald's formula.

**Table 5:** Effect of most effective dose of *Trichosanthes dioica* fruit aqueous extract on TP and Hb of severely diabetic rats (mean ± S.D.).

Experimental animals	Treatment (aqueous extract)	Pre-treatment levels	Post-treatment levels			
			7 days	14 days	21 days	28 days
Total protein (mg dL^−1^)						
Normal (control I)	D W	8.9 ± 1.8	8.7 ± 1.2	8.5 ± 1.4	8.8 ± 2.1	8.9 ± 2.2
SD (control II)	D W	5.6 ± 2.6	5.1 ± 2.5	5.3 ± 1.7	5.7 ± 2.3	5.6 ± 1.5
SD (treated III)	1000 mg kg^−1^	5.4 ± 2.2	5.6 ± 2.4*	6.4 ± 2.2*	7.1 ± 1.9*	7.4 ± 2.1*

Total hemoglobin (g dL^−1^)						
Normal (control I)	D W	13.8 ± 2.5	13.8 ± 2.5	14.2 ± 2.5	14.0 ± 2.5	14.3 ± 2.5
SD (control II)	D W	10.3 ± 3.1	10.4 ± 2.5*	10.2 ± 3.7	10.7 ± 2.5	10.1 ± 2.5
SD (treated III)	1000 mg kg^−1^	11.3 ± 2.7	12.0 ± 2.6*	14.1 ± 2.2**	13.2 ± 2.5*	13.1 ± 2.8*

*P< 0.05, **P< 0.01 compared to pretreatment levels.

**Table 6:** Effect of most effective dose of *Trichosanthes dioica* fruit aqueous extract on US and bw of severely diabetic rats (mean ± S.D.).

Experimental animals	Treatment (aqueous extract)	Pre-treatment levels	Post-treatment levels			
			7 days	14 days	21 days	28 days
Urine Sugar						
Normal (control I)	D W	Nil	Nil	Nil	Nil	Nil
SD (control II)	D W	+++	+++	++++	++++	++++
SD (treated III)	1000 mg kg^−1^	++++	++++	+++	++	+

Body weight (g)						
Normal (control I)	D W	200	200	210	210	210
SD (control II)	D W	190	190	180	180	180
SD (treated III)	1000 mg kg^−1^	200	200**	210*	210*	210**

*P< 0.05, **P< 0.01 compared to pretreatment levels.

## List of Abbreviations

BGL: Blood glucose level
bw: Body weight
DW: Distilled water
FBG: Fasting blood glucose
GTT: Glucose tolerance test
h: Hours
Hb: Haemoglobin
HDL: High density lipoprotein
Kg: Kilogram
LD_50_: Lethal dose 50%
LDL: Low density lipoprotein
Mg: Milligram
PPG: Postprandial glucose
S.D.: Standard deviation
STZ: Streptozotocin
TC: Total cholesterol
TP: Total protein
TG: Triglycerides
US: Urine sugar.
